# A novel nonsense mutation in keratin 10 causes a familial case of recessive epidermolytic ichthyosis

**DOI:** 10.1002/mgg3.6

**Published:** 2013-04-15

**Authors:** Jeydith A Gutierrez, Zeina C Hannoush, Luis G Vargas, Allison Momany, Carmen C Garcia, Jeffrey C Murray, Martine Dunnwald

**Affiliations:** 1 Internal Medicine Department, Carver College of Medicine, University of IowaIowa City, Iowa; 2 Escuela de Medicina Luis Razetti, Universidad Central de VenezuelaCaracas, Venezuela; 3 Department of Pediatrics, Carver College of Medicine, University of IowaIowa City, Iowa; 4 Cátedra de Patología General y Fisiopatología, Instituto de Medicina Experimental, Universidad Central de Venezuela (UCV)Caracas, Venezuela

**Keywords:** Bullous congenital ichthyosiform erythroderma, epidermolytic hyperkeratosis, epidermolytic ichthyosis, genetics, keratin, keratin 10, mutations, nonsense mediated mRNA decay

## Abstract

Epidermolytic ichthyosis (EI) is a rare skin disorder characterized by generalized erythroderma and cutaneous blistering at birth, which is substituted by hyperkeratosis later in life. It is caused by autosomal dominant mutations in highly conserved regions of *KRT1* and *KRT10*. To date, only four mutations with autosomal recessive inheritance of EI have been described in consanguineous families. All of them affect the 2B domain of *KRT10*. In the present study, we describe four patients with EI (including one lethal case) born from unaffected parents in a consanguineous family of a native Venezuelan community. The objective of this study was to characterize the clinical, genetic, and morphological aspects of the disease in this family, as well as understand its functional implications. Genomic DNA was sequenced for *KRT10* and *KRT1*. Immunofluoresence for keratin expression was performed on cutaneous biopsies. After examination of cutaneous biopsies histology, our results showed hyperkeratosis and acantholysis with an expanded granular layer. Sequencing of *KRT10* demonstrated a nonsense mutation (p.Tyr282Ter.) corresponding to the 1B domain of the protein in patients and a heterozygous pattern in other family members, resulting in complete absence of K10. The loss of K10 was compensated by upregulation of K14 and K17. In conclusion, this novel mutation in *KRT10* is the first recessive genetic variation that is not located in the so called “hot spot” for recessive EI, suggesting that other areas of the gene are also susceptible for such mutations.

## Introduction

Epidermolytic ichthyosis (EI), formerly known as epidermolytic hyperkeratosis or bullous congenital ichthyosiform erythroderma (BCIE) (Oji et al. 2010), is a skin disease that affects one in 200,000 newborns (Muller et al. 2006). Clinically, it is characterized by generalized widespread cutaneous blistering and erythema at birth. Neonates are at risk of developing infections and electrolytic disorders, which may lead to death. Blistering improves with age and is replaced by progressive hyperkeratosis (Kwak and Maverakis 2006).

EI has been classically described as an autosomal dominant disease, caused by a variety of mutations in the coexpressed keratin genes *KRT1* and *KRT10* (Arin et al. 2011). The functional consequence is impaired network of tonofilaments in suprabasal keratinocytes, leading to keratin clumping and cytolysis (Muller et al. 2006). More than 100 mutations have been associated with EI, most of which are heterozygous missense mutations located in highly conserved helix boundary motifs (Arin et al. 2011). More recently, rare cases of recessive EI have been reported affecting *KRT10*, leading to a complete absence of the protein (Muller et al. 2006; Tsubota et al. 2008; Terheyden et al. 2009; Covaciu et al. 2010). Our study identifies a novel recessive mutation in a unique region of *KRT10* in four patients of a consanguineous family of a native Venezuelan community.

## Materials and Methods

**Figure 1:**
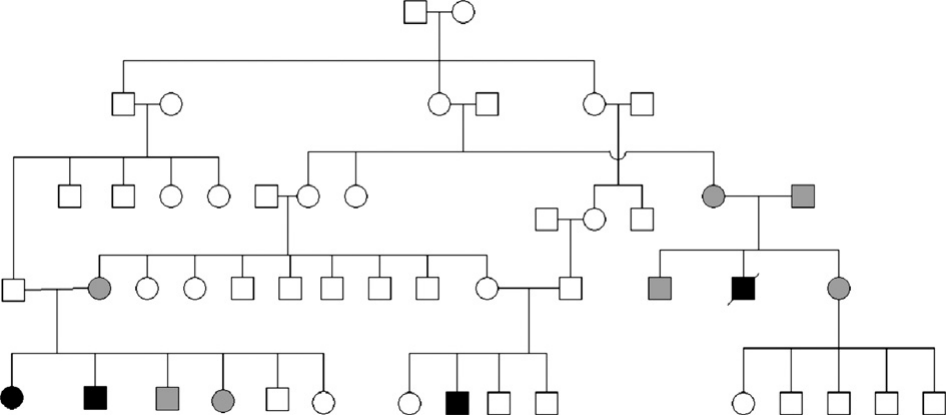
Familial pedigree. Three members in the last generation were affected (black) and one patient was deceased in the previous generation (black, cross). Confirmed heterozygote carriers of the mutation (gray).

**Figure 2:**
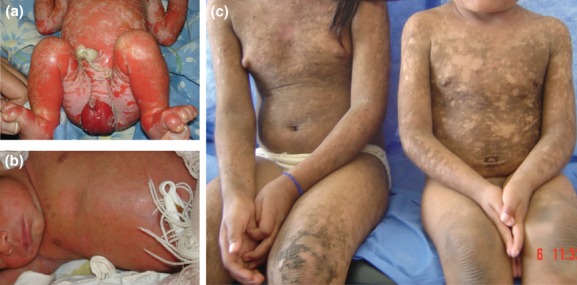
Clinical features. (a) Clinical presentation of one of the epidermolytic ichthyosis (EI) patients at birth. Note the generalized erythroderma, blistering, and erosions. (b) Patient shown in b a few weeks later; note the healing of the erosions, a mild hyperkeratosis, and the absence of blistering. (c) Affected siblings (from left to right, 12 and 7 years old) showing generalized hyperkeratosis.

In this study, we describe a familial case of EI with autosomal recessive inheritance observed in an isolated native community in Venezuela. Affected individuals included two siblings and a cousin in the maternal line, all born from unaffected consanguineous parents (Fig. 1). A fourth case (lethal) occurred in the previous generation according to the history provided by the family. This patient died in the neonatal period due to complications of the disease. All the patients presented with generalized erythema, severe cutaneous blistering, and erosions at birth (Fig. 2A). Similar manifestations have been previously described in recessive as well as dominant EI cases (Chassaing et al. 2006; Terheyden et al. 2009). Generalized hyperkeratosis developed later in life (Fig. 2B and C). None of them showed involvement of the palmoplantar areas, consistent with previous observations with *KRT10* mutations, including all the recessive EI cases (Terheyden et al. 2009).

**Figure 3:**
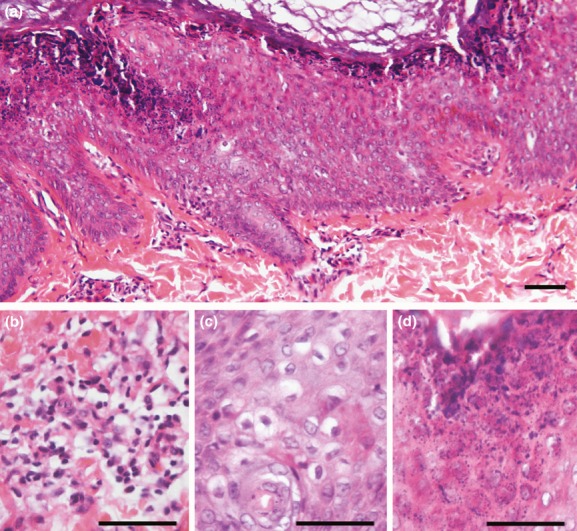
Histopathological findings. (a) Histology of cutaneous forearm samples indicate compact hyperkeratosis. (b) Presence of dermal inflammatory infiltrates. (c) Acanthosis with vacuolar degeneration of suprabasal keratinocytes. (d) Expanded granular layer with increased number of coarse keratohyaline granules. Scale bar = 50 μm.

The study was conducted following the standards of the Helsinki Declaration. Informed consent was obtained from all patients and legal guardians, as well as approval of the Institutional Review Board of Universidad Central de Venezuela and the University of Iowa. Genomic DNA from three EI patients, as well as eight nonaffected family members was isolated from peripheral blood by standard methods. For mutation analyses of *KRT1* and *KRT10*, direct sequencing of polymerase chain reaction (PCR)-amplified fragments using exon–exon specific primer pairs was conducted as described before (Tsubota et al. 2008). Under local anesthesia, skin biopsies of the patients were obtained and analyzed by routine histology (Fig. 3). Immunostaining of control (foreskin) and affected sample was performed using antibodies against K14 (clone LL002, Serotec, Raleigh, NC), K10 (PRB-159P, Covance, Princeton, NJ), K17 (gift from Dr. Coulombe McGowan and Coulombe 1998), and K1 (clone LHK1, Abcam, Cambridge, MA).

## Results and Discussion

**Figure 4:**
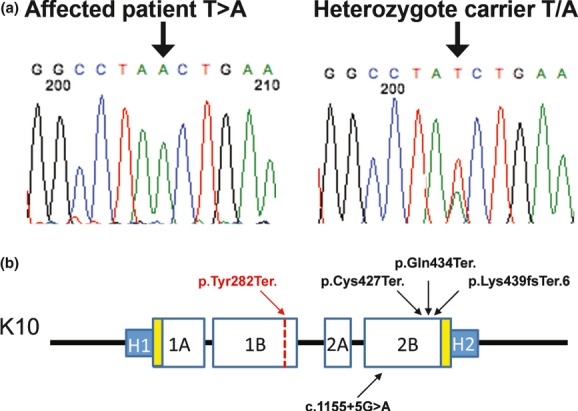
Mutation localization in the context of K10 protein structure. (a) Exon 3 mutation showing homozygous T→A mutation (p.Tyr282Ter.) and a heterozygous carrier. (b) Schematic representation of K10 protein structure. Previous recessive epidermolytic ichthyosis (EI) mutations in the 2B domain are denoted in black. p.Tyr282Ter. (red) is located in the 1B domain of the protein.

A homozygous point mutation (T→A) at position 846 in exon 3 of *KRT10* was found in the three EI patients (Fig. 4A). All the unaffected family members (including both parents of the deceased patient) showed a heterozygous pattern (A/T) at this locus (Fig. 4A). This novel mutation leads to a change from the TAT codon at position 282 encoding a Tyrosine residue to a TAA stop codon (p.Tyr282Ter.) in the 1B domain of the protein. This genetic variant was not found in the “thousand genome” database consisting of 1000 unrelated individuals nor in the exome variant database (http://evs.gs.washington.edu/EVS/) of more than 6500 individuals, and is therefore not a common polymorphism or even rare known variant (Consortium 2010). Previously reported recessive EI cases were due to nonsense mutations resulting in premature termination codon (PTC) in exon 6 of *KRT10*. All these mutations, even the most recently described involving the splice site in intron 5 (Covaciu et al. 2010) were located in close proximity in the 2B domain of the protein. Therefore, this area was proposed as a “genetic hotspot” for recessive EI (Fig. 4B) (Terheyden et al. 2009). Our genetic variant is in exon 3, which, as of to date, makes this mutation the only recessive variant affecting a different area of the protein. The presence of clinically unaffected heterozygous carriers suggests that one allele of the gene is sufficient to retain a normal phenotype. No mutation in *KRT1* was found.

**Figure 5:**
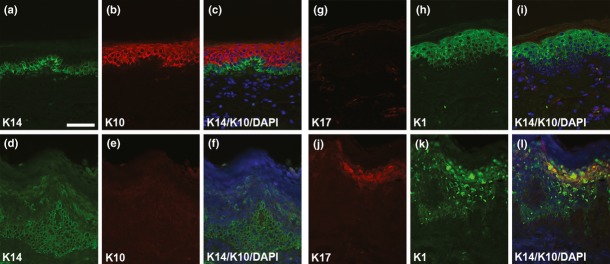
Keratin expression. Immunofluorescence staining of control (foreskin, a–c, g–i) and affected samples epidermolytic ichthyosis (EI, d–f, j–l) for K14 (a, d), K10 (b, e), K17 (g, j), and K1 (h, k). In EI samples, note the absence of K10. Ectopic expression of K14 in the suprabasal layers and expression of K17 in the interfollicular epidermis may compensate for the loss of K10. Also, note the clumpy appearance of K1 in the upper spinous and granular layers. Scale bar = 50 μm.

PTC could result in truncated gene products or lead to nonsense-mediated mRNA decay (NMD). Normally exon-junction protein complexes are displaced by the translation machinery as the elongation of the transcript occurs (Silva and Romao 2009). When one of these complexes remains associated with the mRNA, NMD occurs (Muller et al. 2006; Terheyden et al. 2009). The distance between the PTC and the 3'-most exon–exon junction determines the possibility to elicit NMD, and varies between genes. For keratins, mRNAs with PTC located at least 92 nucleotides upstream of the 3’-most exon–exon junction are expected to undergo NMD. Previously reported cases of recessive EI with PTC located 461, 476, and 497 nucleotides upstream of the 3’-most exon–exon junction demonstrated NMD of K10 (Terheyden et al. 2009). Therefore, the p.Tyr282Ter. mutation, located at an increased distance from the 3’-most exon–exon junction, would be expected to undergo NMD as well. We confirmed this hypothesis by performing immunofluorescence of paraffin-embedded skin sections (Fig. 5). Our results show the complete absence of K10 in the affected patients (Fig. 5E and F). Furthermore, we observed aggregated K1 expression and ectopic expansion of K14 in the suprabasal layers (Fig. 5D, F, K, and L). Additionally, the presence of K17 in the interfollicular epidermis where K1 shows aggregates (Fig. 5J–L) suggests a compensatory mechanism from other keratins for the loss of K10, in agreement with previous reports (Terheyden et al. 2009).

Our study presents a novel mutation responsible for an autosomal recessive EI affecting the 1B domain of K10. The identification of mutations involved in recessive EI is critical for genetic counseling especially in isolated communities with high rate of consanguineous marriages and low genetic flow.
